# Circulating Tumor DNA in Early and Metastatic Breast Cance—Current Role and What Is Coming Next

**DOI:** 10.3390/cancers16233919

**Published:** 2024-11-22

**Authors:** Christian Martin Tegeler, Andreas Daniel Hartkopf, Maggie Banys-Paluchowski, Natalia Krawczyk, Tanja Fehm, Bernadette Anna Sophia Jaeger

**Affiliations:** 1 Department of Obstetrics and Gynecology, University Hospital Tübingen, 72076 Tübingen, Germany; 2 Department of Peptide-Based Immunotherapy, Institute of Immunology, University Hospital Tübingen, 72076 Tübingen, Germany; 3 Department of Gynecology and Obstetrics, University Hospital Schleswig-Holstein, Campus Luebeck, 23538 Luebeck, Germany; 4 Department of Gynecology and Obstetrics, University Hospital Düsseldorf, 40225 Duesseldorf, Germany; 5 Center for Integrated Oncology (CIO) ABCD, 40225 Duesseldorf, Germany

**Keywords:** ctDNA, liquid biopsy, early breast cancer, metastatic breast cancer, prognostic marker, predictive marker

## Abstract

The progress that has been made in recent years in relation to liquid biopsies in general and circulating tumor DNA (ctDNA) in particular can be seen as groundbreaking for the future of breast cancer treatment, monitoring and early detection. Cell-free DNA (cfDNA) consists of circulating DNA fragments released by various cell types into the bloodstream. A portion of this cfDNA, known as ctDNA, originates from malignant cells and carries specific genetic mutations. Analysis of ctDNA provides a minimally invasive method for diagnosis, monitoring response to therapy, and detecting the emergence of resistance. Several methods are available for the analysis of ctDNA, each with distinct advantages and limitations. Quantitative polymerase chain reaction is a well-established technique widely used due to its high sensitivity and specificity, particularly for detecting known mutations. In addition to the detection of individual mutations, multigene analyses were developed that could detect several mutations at once, including rarer mutations. These methods are complementary and can be used strategically depending on the clinical question. In the context of metastatic breast cancer, ctDNA holds particular promise as it allows for the dynamic monitoring of tumor evolution. Through ctDNA analysis, mutations in the *ESR1* or *PIK3CA* genes, which are associated with therapy resistance, can be identified. This enables the early adjustment of treatment and has the potential to significantly enhance clinical outcome. The application of ctDNA in early breast cancer is an ongoing investigation. In (neo)adjuvant settings, there is preliminary data indicating that ctDNA can be used for therapy monitoring and risk stratification to decide on post-neoadjuvant strategies. In the monitoring of aftercare, the detection of ctDNA appears to be several months ahead of routine imaging. However, the feasibility of implementing this approach in a clinical setting remains to be seen. While the use of ctDNA as a screening method for the asymptomatic population would be highly advantageous due to its minimally invasive nature, the available data on its clinical benefit are still insufficient. Nevertheless, ctDNA represents the most promising avenue for fulfilling this potential future need.

## Simple Summary

Liquid biopsy, particularly involving the detection of circulating tumor DNA (ctDNA), has emerged as a promising tool in breast cancer management. Unlike traditional tissue biopsies, ctDNA provides a non-invasive method by detecting DNA fragments released by tumor cells into the bloodstream. Detection techniques include PCR-based methods, targeted panels for known mutations, and personalized assays based on the individual tumor profile. In early-stage breast cancer, ctDNA shows potential for assessing response to treatments like chemotherapy and identifying patients at elevated risk of recurrence. However, ctDNA detection in early-stage disease remains challenging due to low tumor DNA concentrations in blood. In metastatic breast cancer, ctDNA is utilized to monitor disease progression, evaluate treatment response, and detect emerging resistance mutations, enabling timely adjustments in therapy. Although ctDNA holds significant potential for enhancing personalized care, further research is necessary to validate its role in routine clinical practice for comprehensive breast cancer management.

## 1. Introduction

With the National Decade against Cancer, the German Federal Ministry of Education and Research has been pooling efforts in the fight against tumor diseases since 2019. The defined goals are strong cancer research, rapid transfer of research results into practice, improved prevention, and early detection. This shows both the necessity and the seriousness with which the increasing number of cancer cases in Western countries is being taken. In recent years, the introduction of liquid biopsy has constituted a substantial advance in the field of oncology. In comparison to a conventional biopsy of tumor tissue, liquid biopsy offers a minimally invasive approach to gain certain information about a tumor and enables, e.g., the detection of tumor cells or fragments of tumor cells in the blood. One main focus lies in the detection of circulating tumor DNA (ctDNA). Tumor cells release fragments of their DNA into the circulation, which can be detected as ctDNA using highly sensitive methods. However, clinical utilization is (still) limited. In the context of metastatic breast cancer, ctDNA is employed to monitor disease progression. As metastatic cells disseminate and undergo evolutionary changes, they frequently acquire novel mutations that can be monitored through ctDNA analysis. The application of ctDNA in early breast cancer is still an area of active research. Here, we reviewed the current status of ctDNA in early and metastatic breast cancer as well as potential future applications.

## 2. Liquid Biopsy and the Role of ctDNA

In recent years, the introduction of liquid biopsy has been a significant development in oncology. In comparison to a conventional biopsy of tumor tissue, liquid biopsy offers a minimally invasive approach to gain certain information about a tumor. Liquid biopsy is used to detect tumor cells or fragments of tumor cells. Initially, the focus was on detecting circulating tumor cells (CTCs), which had detached from the primary tumor or a metastatic lesion and could be identified in the peripheral blood [1]. However, this detection method was not without limitations. While the cells were often informative about the tumor, they were not always detectable, even in patients with metastatic disease [2]. Consequently, there has been a desire for more precise examination methods.

CfDNA refers to extracellular DNA molecules that are generated from various cell types. Tumor cells release fragments of their DNA into the circulation [3], which can be detected as ctDNA using highly sensitive methods. In cancer patients, one fraction of the circulating cfDNA is ctDNA, accounting for 0.01–50% of the total cfDNA in most cases [4]. The cell-specific somatic genomic alterations permit the differentiation of tumor-derived ctDNA from normal cfDNA. Depending on the methodology used, the detection of ctDNA is more prevalent than that of CTCs [4]. The isolation of ctDNA was first demonstrated several years ago. The initial analyses in humans were constrained by the limitations of individual mutations, particularly TP53. A significant challenge was the high detection limit, which precluded the use of this method in clinical settings. Nevertheless, the first studies could already provide insights into the dynamics of tumor disease. In the years that followed, ctDNA became more established and the diagnostic measures were significantly refined [5,6]. In addition to the detection of individual mutations, multigene analyses were developed that could detect several mutations at once, including rarer mutations [7].

Another significant achievement was the development of drugs that are effective against specific mutations, thereby overcoming resistance mechanisms (e.g., ESR1 or PIC3CA mutations). The specific targeting of these mutations has further enhanced the therapeutic landscape in breast cancer, leading to regular analysis of these mutations based on their relevance to therapy [8,9].

Therefore, ctDNA is a promising tool to monitor treatment response and detect disease relapse [10]. Furthermore, ctDNA can be used not only to monitor tumor load but also to identify treatment targets [11]. One of the primary benefits of ctDNA is its ability to capture the heterogeneity of tumors, including metastatic lesions that may not be fully represented by a single tissue biopsy.

## 3. Methods for ctDNA Detection and Analysis

ctDNA analyses range from limited polymerase chain reaction (PCR)-based approaches to next-generation sequencing technologies. The initial detection methods employed PCR to identify mutated genes in the blood of affected patients [12]. These methods were highly specific, but could only detect one mutation at a time. Furthermore, the detection limit was too high to draw any conclusions outside of clinical trials. Consequently, methods were developed that were significantly more sensitive. Next-generation sequencing technologies offer a significant advantage over traditional sequencing methods. For instance, they enable the sequencing of a large number of genes, including the entire exome, providing more comprehensive information on the tumor [13].

For the analysis of ctDNA, there are a variety of tests, some of which are commercially available. Generally, there are three approaches for the detection of ctDNA in breast cancer:

(I) Untargeted approaches use whole-genome or whole-exome sequencing techniques to discover new mutations in tumor DNA and are therefore suitable to monitor tumor heterogeneity or to find new drug targets and are predominately used in patients with metastatic disease. However, sensitivity is lower, and untargeted approaches are more expensive [14].

(II) Targeted approaches use a panel of known driver, druggable, or resistance mutations (i.e., hotspot mutations in the *ESR1* or *PIK3CA* gene) or cover a small number of complete driver gene sequences. This is especially useful to identify patients for a certain targeted therapy (i.e., elacestrant or alpelisib), to monitor its efficacy and to identify emerging resistance mutations [9,15].

(III) A third approach uses information from primary tumor tissue to design personalized targeted sequencing panels that enable the tracking of patient-specific mutations in plasma [16,17,18,19]. This approach achieves the highest sensitivity and specificity [20], making it a highly promising technique for early detection of disease recurrence in cases where bulk tumors are not yet visible by conventional imaging methods (see Table 1).

One of the principal distinctions between the various methodologies is the number of mutations that can be identified. While untargeted approaches are capable of detecting a considerable number of mutations, targeted tests or tumor-informed tests are designed to identify single or multiple tumor-specific mutations. Nevertheless, even single mutations, which can be identified at a relatively low cost, can have a significant impact on patient treatment. In light of the growing financial pressures within the healthcare system, it is crucial to conduct a thorough evaluation of the necessity for broad patient testing with untargeted approaches. One notable advantage is the decline in the costs associated with new methods, e.g., whole-exome sequencing, in recent years. However, the usage of these approaches in clinical practice remains constrained, particularly because the outcome is not indicative of a therapeutic consequence. Additionally, the high sensitivity of these methods requires specialized equipment and expertise, which further limits their widespread implementation. However, ongoing technological advancements and cost reductions hold promise for broader accessibility in the future. Table 1 presents a simplified overview of the principal distinctions between the individual methods.

## 4. Metastatic Breast Cancer—ctDNA Current Role and What Is Coming Next in the Course of the Chronic Disease

ctDNA has become an essential tool in the understanding and management of metastatic breast cancer [3,8,9,10,21]. This technology facilitates the detection of genetic mutations and alterations, providing a real-time picture of the disease’s genetic landscape. In terms of metastasis, ctDNA is particularly valuable as it can capture the heterogeneity of metastatic lesions that might not be fully represented by a single tissue biopsy [21].

Currently, ctDNA in metastatic breast cancer is used to monitor disease progression and clonal evolution. As metastatic cells spread and evolve, they often acquire new mutations that can be tracked through ctDNA analysis [22]. Ongoing monitoring allows for the early detection of metastasis and changes in tumor biology, enabling timely and precise adjustments to treatment plans. For instance, *ESR1* gene alterations, particularly mutations in the ligand-binding domain of the estrogen receptor, are a significant concern in metastatic breast cancer [23]. ctDNA analysis helps in identifying specific genetic alterations associated with poor prognosis, such as mutations in the *PIK3CA* gene or the aforementioned alterations in the *ESR1* gene, which are known to drive resistance to hormonal therapies in breast cancer [24,25]. These mutations can activate the estrogen receptor independently of its ligand, leading to continued tumor growth despite antihormonal therapies designed to block estrogen production or its receptor. When such mutations are detected early, clinicians can adjust treatment strategies accordingly. For example, newer drugs such as next-generation selective estrogen receptor degraders are more effective against tumors harboring these specific mutations [8].

Compared to traditional tissue biopsies, ctDNA monitoring in metastatic breast cancer offers a significant advantage, as it reduces the need for invasive tissue biopsies, which is particularly beneficial for patients with hard-to-reach tumors or for those who would be burdened by repeated biopsies [4,26]. Additionally, ctDNA may capture mutations that might be missed in a single-site tissue biopsy. This method also allows for more frequent monitoring, enabling real-time assessment of treatment response and disease progression. Although this approach is already justified in metastatic breast cancer, the measurement of ctDNA cannot yet replace the classic tumor biopsy for specific questions regarding tumor biology at primary diagnosis.

In the future, ctDNA may be further utilized for disease management and risk assessment. Rising levels of ctDNA may indicate the development of new metastatic sites, even before these changes become apparent through imaging techniques. Specific ctDNA markers might provide insights into changing tumor biology, thereby improving the long-term planning and monitoring of treatment. Current studies are investigating the potential applications of ctDNA in the monitoring of metastatic breast cancer. For instance, the interventional MONDRIAN study (NCT04720729) uses a personalized test based on tumor mutations to detect alterations in ctDNA levels by digital-droplet PCR during an initial cycle of chemotherapy and determine whether the current regime should be continued or if a different drug should be employed. The NCT05826964 trial involves patients with metastatic hormone receptor-positive, human epidermal growth factor receptor 2-negative breast cancer, who are exhibiting increasing ctDNA levels under aromatase inhibitor and cyclin-dependent kinase 4/6 (CDK4/6) inhibitor therapy. ctDNA levels are tested frequently by a non-tumor-informed test searching for multiple mutations and as soon as they are rising, these patients are switched early to another drug combination. Additionally, trials are currently investigating the comparability of invasive tumor biopsy with liquid biopsy, with the objective of further refining the latter (NCT04962529, NCT05919212). All of these approaches can ultimately lead to more personalized and effective treatment regimens for patients with metastatic breast cancer.

## 5. Early Breast Cancer—The Current and the Future Role of ctDNA During (Neo)Adjuvant Treatment

### 5.1. Challenges in the Detection of ctDNA in Early Breast Cancer

In early-stage breast cancer, the detection of ctDNA is difficult due to its low concentration. Further, the data have long been heterogeneous due to the utilization of different test methods. Nevertheless, it is highly desirable to introduce ctDNA as an early and non-invasive detection method that could be implemented for diagnosis, therapy monitoring, and prediction of prognosis as well as recurrence risk. Repeated blood sampling at defined time points would enable this easy-to-use monitoring method compared to other, more invasive or time-consuming procedures [27].

The main limitation of the initial studies employing ctDNA in early breast cancer is the tracking of only one or a few mutations in plasma samples, for example, TP53 [28,29]. Other relevant mutations were not identified using this method, resulting in underestimation and false-negative detection of ctDNA. The actual challenge—as mentioned above—is to decide whether to use a non-tumor-informed (II) or a tumor-informed (III) detection assay (Table 1). Both approaches focus on several mutations (leading to more accurate results) by using either a predefined panel including well-known common cancer mutations or a cancer-specific panel after identifying the actual mutational status of the corresponding primary tumor. Especially in triple-negative breast cancer (TNBC), non-tumor-informed tests are more precise due to the heterogeneous mutation status of TNBC compared to other subtypes [30]. It is also worth noting that the proportion of ctDNA-positive patients was higher in TNBC and HER2+ subtypes, for example, in the neoadjuvant I-SPY2 trial, and ctDNA positivity was additionally related to tumor size [31]. In this phase II trial, a personalized ctDNA test was designed to detect up to 16 patient-specific mutations (from whole-exome sequencing of pre-treatment tumors). Regarding the time of detection, which must also be defined and discussed in the context of NAT, blood was taken at four different times in this study (before treatment, three weeks after the start of paclitaxel treatment, between paclitaxel and anthracycline treatments, or before surgery). 

### 5.2. Prediction of Therapy Response and Prognosis

Aiming to monitor neoadjuvant chemotherapy (NAT) and guide therapy decisions, the applied test must be precise, reliable, and reproducible. Initial reviews that summarize the current state of knowledge on the detection of ctDNA during NAT show that a decrease in detected ctDNA levels during NAT is a good prognostic marker in terms of relapse-free survival [32,33]. Even worse prognosis due to a non-pathologic complete response (non-pCR) after NAT is lessened by ctDNA negativity at this time point in the I-SPY 2 study. It is noteworthy that just three weeks after the start of NAT, a relevant proportion of patients with serial blood draws were cleared at this time point (20/58) [31]. Correspondingly, persistent ctDNA positivity was related to non-pCR. The detection of ctDNA was investigated as a biomarker for therapy response with a total of 84 early-stage breast cancer patients at high risk of metastatic relapse. In 73% of the patients, ctDNA was detectable before the start of treatment and decreased during treatment to 9% after completion of NAT. Of note, the positive predictive value (PPV) of ctDNA positivity in foreseeing failure to achieve pCR increased over time, indicating that ctDNA analysis after completion of NAT can be used to stratify patients by risk and plan post-neoadjuvant treatments [31].

On the other hand, in the NeoALTTO trial, ctDNA negativity at baseline in patients with HER2-enriched breast cancer was associated with the highest pCR rates compared to other subtypes, suggesting that these patients may be good candidates for de-escalation strategies [29]. Plasma DNA was collected in this phase III trial before NAT, 2 weeks after the start of treatment, and before surgery. ctDNA was assessed using digital PCR for PIK3CA and TP53 mutations.

In addition to the correlation with response to treatment, several studies have shown that ctDNA positivity is a negative prognostic marker in early-stage breast cancer. In Nader-Marta’s recent meta-analysis of 57 studies involving a total of 5779 patients, ctDNA positivity was associated with poorer disease-free survival (DFS) before NAT (hazard ratio (HR) 2.98, 95% confidence interval (CI) 1.92–4.63), after NAT (HR 7.69, 95% CI 4.83–12.24), and during follow-up (HR 14.04, 95% CI 7.55–26.11). Accordingly, the detection of ctDNA was associated with poorer overall survival (OS) at all time points (before NAT: HR 2.76, 95% CI 1.60–4.77; after NAT: HR 2.72, 95% CI 1.44–5.14; and during follow-up: HR 9.19, 95% CI 3.26–25.90) [34].

### 5.3. ctDNA Compared to CTCs as a Prognostic Marker in Early Breast Cancer

The combined detection of ctDNA and CTCs was explored in the post-neoadjuvant BRE12-158 study. In this phase II trial, the detection of ctDNA was significantly associated with poorer distant disease-free survival (DDFS), DFS, and OS in 196 TNBC patients with non-pCR. FoundationACT or FoundationOne liquid assays (Foundation Medicine Inc., Boston, MA, USA, both tumor-non-informed) were used to sequence ctDNA at day 1 or at the first round of post-neoadjuvant treatment. CTCs were screened using an epithelial cell adhesion molecule-based microfluidic positive selection device. However, the combination of ctDNA and CTCs provided additional information and led to increased sensitivity and discriminatory power. Patients who were both ctDNA-positive and CTC-positive had a significantly worse DDFS at 24 months than patients who were ctDNA-negative and CTC-negative (52% vs. 89%, respectively, compared to 56% vs. 81% in ctDNA-only-positive vs. ctDNA-only-negative patients, respectively) [35]. Besides the identification of ctDNA in patients with TNBC, Ortolan et al. investigated the detection of CTCs using the marker-independent Parsortix approach for CTC enrichment in combination with positive and negative selection with the DEPArray. They came to the conclusion that CTCs are frequently non-conventional (i.e., non-epithelial) in most recurrence cases and would not have been detected with any of the commercially available epithelial marker approaches, including *Cell Search* [36]. This should be considered in further studies on CTCs during follow-up and when comparing their significance with that of ctDNA.

### 5.4. ctDNA as Possible Marker for Therapy Decision

The circumstance that ctDNA positivity at any of the above time points is associated with poor outcome [34] raises the question of early treatment switch or escalation of post-neoadjuvant therapy regimens [32]. The most frequently altered, druggable gene mutations detected in ctDNA are *PTEN*, *PIK3CA*, *ESR1*, *AKT*, and *HER2* [32]. If targeted therapy options are available (see above metastatic setting), and the respective mutation is detected, it is conceivable to apply these drugs under study conditions even in early disease as practiced before with several other agents. In the case of a lack of liquid biopsy-based targeted therapies, other strategies must be discussed. Escalating or an early switch to standard chemotherapy as well as extending the indication for established post-neoadjuvant therapies are possible alternatives. Poly-ADP ribose polymerase (PARP) or CDK4/6 inhibitors could be used according to their spectrum of action in high-risk situations defined by ctDNA positivity after NAT, alongside the known clinic–pathologic factors. Further research and clinical trials addressing these questions are needed. Table 2 summarizes current clinical trials.

### 5.5. ctDNA During Follow-Up—Will the Current Standard Be Changed Soon?

The current routine in follow-up care for early breast cancer is physical examination and annual mammography. Laboratory tests and advanced imaging are not recommended due to the lack of clinical benefit. Given the clinical need to identify biomarkers that predict recurrence risk, ctDNA appears to be the most promising. Despite the necessity for further studies in this area, ctDNA remains the most encouraging option that will hopefully allow us to meaningfully differentiate between patients who are definitively cured or highly likely to be cured and those who have a very high risk of recurrence [37]. Nevertheless, the question of the most appropriate test method is also being discussed in relation to the follow-up phase. Ortolan et al. performed ctDNA analysis restricted to the known mutation profile of the primary tumor (tumor-informed). One disadvantage is that clonal evolution cannot be studied and ctDNA might be missed [36]. On the other hand, focusing on the known mutation profile in the tumor reduces the risk of false-positive results, especially in consideration of recent reports of somatic plasma mutations emerging from clonal hematopoiesis [38]. However, sequencing of matching buffy coat samples could be used to exclude germline mutations and variants arising at clonal hematopoiesis of indeterminate potential (CHIP) from analysis [31].

The group of Zhou analyzed the value of ctDNA with respect to sampling time points and geographic regions regarding the recurrence of different cancer types (breast cancer, non-small cell lung cancer, gastric cancer, colorectal cancer, renal cancer, esophageal cancer, melanoma, and bladder cancer) treated with NAT. The results showed that ctDNA detection was associated with recurrence in breast and digestive tract cancer. However, a strong correlation was noticed only in the case of ctDNA positivity at the time of post-neoadjuvant treatment and post-surgery but not in the case of pre-neoadjuvant detection [39]. La Rocca et al. on the other hand evaluated current follow-up practices and the value of ctDNA monitoring in the surveillance of high-risk breast cancer patients treated at a comprehensive cancer center with curative intent. Five recurrent cases were identified by intensive follow-up, five by symptoms and two incidentally. ctDNA was detected prior to disseminated disease in all evaluable cases apart from two cases with bone-only and single liver metastases. The median time between the detection of ctDNA and suspicious imaging findings was 3.81 months (SD, 2.68) and 8 months (SD, 2.98) until final recurrence diagnosis. ctDNA was untraceable in the absence of disease and in two suspected cases that were later unconfirmed [40]. In their analyses of TNBC patients, Ortolan et al. describe similar results: in 83% of analyzed cases, detection of ctDNA preceded clinical diagnosis of distant metastases by 8.9 months (range, 6.5–13.1 months), showing excellent specificity [36]. The systematic review and meta-analysis of Nader-Marta et al. confirmed the above findings. They evaluated 14 studies in which ctDNA was detected during the follow-up period with regard to DFS and OS and showed that the risk of recurrence and mortality is particularly higher when ctDNA is detected after neoadjuvant or adjuvant therapy using tumor-informed assays. The pooled HRs were numerically higher in the case of ctDNA detection during the follow-up period compared to baseline. It is noteworthy that the presence of ctDNA precedes the diagnosis of overt metastases by an average of 10.81 months with a specificity of 70% up to 100% [34]. Therefore, it is conceivable that ctDNA not only represents a further prognostic factor but is also capable of predicting disease recurrence even at the level of the individual patient. The identified “lead time” could provide a unique window of opportunity for the application of non-cross-resistant therapies with the aim of preventing overt clinical recurrence [36]. It remains to be seen whether this can be employed clinically, and if so, to what extent, in order to prolong survival while simultaneously reducing costs.

In the German Survive study (Standard Surveillance vs. Intensive Surveillance in Early Breast Cancer; NCT05658172), the question of optimal follow-up care will be investigated using these new approaches. In addition to routine guideline-based follow-up consisting of clinical examination and imaging, an additional follow-up using liquid biopsy (tumor markers, CTCs, and ctDNA) will be offered in a 1:1 randomization. The intervention phase is planned for 5 years with an additional observation period of 5 years. The primary endpoints of the study are OS and the Overall Lead Time Effect. The study aims to clarify the role of liquid biopsy in follow-up care. In addition, therapy intervention studies will be implemented to which patients can be transferred according to the corresponding indication.

## 6. ctDNA as Screening Method for Asymptomatic Population—Dream or Realistic Future Scenario?

The potential use in the early diagnosis of breast cancer is undoubtedly the most challenging but also the most desirable application of ctDNA, as its detection by non-invasive methods makes it an attractive marker for screening the asymptomatic population [27].

For breast cancer screening in clinical practice, mammography is the established gold standard; however, it is limited to a specific age group of individuals. Currently, breast cancer is mostly diagnosed when symptoms are present. Recently, blood-based tests for multi-cancer early detection (MCED) have been developed for individuals of all ages, allowing the general population to be screened for multiple cancers. In 2018, the first blood-based MCED test results were available, showing a specificity of 99% and a sensitivity of 33% for the detection of eight tumor types, including breast cancer, by targeted cfDNA mutation analysis combined with circulating protein evaluation—the CancerSEEK test [41].

There are many aspects to consider when discussing blood-based MCED testing. To minimize false-positive results, which would cause unnecessary psychological distress and unnecessary radiation doses since they require follow-up examinations, the specificity must be high. Additional regulations for further diagnostics are needed for patients with a positive ctDNA test result but without identifiable tumors on imaging. In this context, it must be pointed out that the specificity of ctDNA can be compromised by the already-mentioned changes in the hematopoietic system during aging. For population screening, the sensitivity needs to be at a sufficient level, as the incidence of cancer in the general population is low. The most important parameter in this context is the PPV, which indicates the probability that a person with cancer will have a positive test result. The PPV depends on the sensitivity, specificity, and disease prevalence in the population being studied. In addition, another restriction is that the clinical utility of MCED tests has not yet been demonstrated. It is not yet known whether these tests shift the time of detection of the disease from a later stage to earlier stages and whether the early diagnosis is early enough to achieve curative treatment, reduce mortality, and prolong survival [42]. Currently, data show that early-stage cancers can only be detected with low sensitivity [43]. Due to the low sensitivity and low PPV as well as the uncertainty about the clinical benefit in terms of reduced mortality, the use of a blood-based MCED test for population screening in clinical routine practice is currently not recommendable [44].

However, some pilot studies have provided preliminary but promising results [45,46] when ctDNA is used in addition to imaging in routine screening. Rodriguez et al. searched for mutations in patients with suspicious mammography findings before they underwent tissue biopsy. Examination of the corresponding tumor tissue revealed *PIK3CA* mutations in 79.3% (23/29) and *TP53* mutations in 34.5% (10/29). One-third of the patients (10/29) also carried plasma mutations in *PIK3CA* and *TP53*, but mostly with very low allele frequency (Afs) (0.01–3.60%), as expected at this early stage of the disease. The same somatic plasma and tumor mutations were found in 8/29 patients. The detection of ctDNA mutations was associated with younger age and more aggressive clinicopathologic features in this patient population [45]. In another prospective study, 152 patients with suspicious mammography or ultrasound findings were included in order to investigate the value of ctDNA as a clinically useful biomarker. A total of 102 patients were diagnosed with early-stage breast cancer (stage I–III), while the remaining 50 patients had benign breast tumors. Plasma samples were taken from the cancer patients before the operation, 2 days and 3 weeks after the operation, and at the end of chemotherapy. With the help of two different gene panels, at least one somatic mutation was detected in almost all of them (35/36), while ctDNA mutations were detected in 19 of them (52.8%) using one panel. With the other panel, at least one tissue mutation was found in all samples analyzed, and 49 (74.2%) had ctDNA mutations in their preoperative plasma samples. By correlating ctDNA results to the corresponding breast imaging-reporting and data system (BIRADS) scores of the imaging to predict the presence of cancer, the authors estimated a PPV of 92.45% (49/53), a sensitivity of 74.24% (49/66), and a specificity of 92% (46/50). These results suggest that ctDNA testing together with imaging could improve the early diagnosis of breast cancer [46].

## 7. Conclusions

In conclusion, ctDNA provides a dynamic and less invasive approach to the management of metastatic breast cancer, offering insights that inform personalized treatment strategies and improve patient outcomes. Its integration into clinical practice represents a significant advancement in the field of precision oncology, enabling more effective and individualized care for patients with metastatic breast cancer. The initial data in neoadjuvant treatment are also encouraging: ctDNA appears to be able to serve as a marker for risk stratification at the individual patient level. Studies are also investigating the use of ctDNA in follow-up care, demonstrating that ctDNA can precede clinical evidence of disease recurrence by several months. This could represent a unique window of opportunity in which future therapeutic interventions could be used to prevent overt metastases. However, it will be some time before solid data on these potential personalized treatment options are available. The use of ctDNA as a marker for screening the asymptomatic population is highly desirable. But there is currently no evidence of clinical benefit, which has so far prohibited its use in this context.

## Figures and Tables

**Table 1:** Overview of the various techniques employed in the detection and analysis of ctDNA.

Approach	Untargeted Approaches	Targeted Approaches	Personalized Targeting
**Method**	WGS/WES	qPCR/dPCR/NGS	dPCR
**Material for ctDNA analysis**	Blood	Blood	Blood
**Match tumor samples**	Not required	Not required	Required
**Amount of ctDNA needed**	High	Low	Low
**Sensitivity**	Low	Very high	High
**Costs per patient**	High	Low	Medium
**Targetable mutations**	High	Single or few	Medium

Abbreviations: WGS, whole-genome sequencing; WES, whole-exome sequencing; qPCR, real-time polymerase chain reaction; dPCR, digital polymerase chain reaction; NGS, next-generation sequencing; ctDNA, circulating tumor deoxyribonucleic acid.

**Table 2:** Clinical studies on post-(neo)adjuvant therapy decisions in the case of post-therapeutic ctDNA positivity in early breast cancer (EBC). Overview of liquid biopsy-based studies in EBC patients with evidence of molecular relapse (detected by ctDNA monitoring) during follow-up (FU). as well as clinical trials on therapy monitoring using ctDNA in metastatic breast cancer (MBC) patients. ER = estrogen receptor. * Active not recruiting. ** Recruitment terminated prematurely.

Study Name	NCT-Number	Phase	Stage	Inclusion Criteria	Intervention	Country/ Region
A Prospective, Phase II Trial Using ctDNA to Initiate Post-operation Boost Therapy After Adjuvant Chemotherapy in TNBC (Artemis)	NCT04803539	II/III	EBC	TNBC, Stadium II-III	Capecitabin + Camrelizumab + Apatinib	China
Atezolizumab + Sacituzumab Govitecan to Prevent Recurrence in TNBC (ASPRIA)	NCT04434040	II	EBC	TNBC, non-PCR (in the breast or lymph nodes) + circulating tumor DNA in the blood	Atezolizumab + Sacitzumab Govitecan	United States
Kadcyla And Neratinib for Interception of HER2+ Breast Cancer With Molecular Residual Disease (KAN-HER2 MRD)	NCT05388149	II	EBC	HER2-positive, non-PCR, MRD after 2–6 cycles of adjuvant T-DM1	Neratinib (together with T-DM1)	Canada
Circulating Tumor DNA Enriched, Genomically Directed Post-neoadjuvant Trial for Patients With Residual Triple Negative Breast Cancer (PERSEVERE) *	NCT04849364	II	EBC	TNBC, non-PCR	Depending on mutation + Capecitabin	United States
Tirzepatide in Patients With Obesity or Overweight Who Have High Risk Early Breast Cancer and Are ctDNA+	NCT06517212	II	EBC	ER+ > 10%, HER2-, node-positive, body mass index > 27 kg/m^2^, ctDNA-positive	Tirzepatide	United States
Efficacy and Safety Comparison of Niraparib to Placebo in Participants With Human Epidermal Growth Factor 2 Negative (HER2-) Breast Cancer Susceptibility Gene Mutation (BRCAmut) or Triple-Negative Breast Cancer (TNBC) With Molecular Disease (ZEST) *	NCT04915755	III	EBC	TNBC with presence of ctDNA or tumor BRCA mutation	Niraparib	United States
A Trial Using ctDNA Blood Tests to Detect Cancer Cells After Standard Treatment to Trigger Additional Treatment in Early Stage Triple Negative Breast Cancer Patients (c-TRAK-TN) **	NCT03145961	II	EBC/FU	TNBC/moderate or high risk	Pembrolizumab, if ctDNA is detected within 12 months during FU	United Kingdom
DNA-Guided Second Line Adjuvant Therapy For High Residual Risk, Stage II-III, Hormone Receptor Positive, HER2 Negative Breast Cancer (DARE)	NCT04567420	II	FU	ER+/HER- high risk	Fulvestrant + Palbociclib	United States
CDK 4/6 Inhibitor, Ribociclib, With Adjuvant Endocrine Therapy for ER-positive Breast Cancer (LEADER)	NCT03285412	II	FU	ER+/HER2- high risk, detectable ctDNA	Endocrine Therapy + Ribociclib	United States
A Randomized Secondary Adjuvant Treatment Intervention Study Comparing Trastuzumab-Deruxtecan to SOC Therapy in EBC Patients with Molecular Relapse (SURVIVE HERoes) *	NCT06643585	III	FU	HER2 positive or HER2 low, positive ctDNA result obtained in the SURVIVE study	Trastuzumab Deruxtecan	Germany
A Trial of Early Detection of Molecular Relapse With Circulating Tumour DNA Tracking and Treatment With Palbociclib Plus Fulvestrant Versus Standard Endocrine Therapy in Patients With ER Positive HER2 Negative Breast Cancer (TRAK-ER)	NCT04985266	II	FU	ER+/HER2- high risk, detectable ctDNA	Fulvestrant + Palbociclib	France
Elacestrant for Treating ER+/HER2- Breast Cancer Patients With ctDNA Relapse (TREAT ctDNA) (TREAT ctDNA)	NCT05512364	III	FU	ER+/HER2- high risk, ctDNA positive	Elacestrant	Belgium
Levels of Circulating Tumor DNA as a Predictive Marker for Early Switch in Treatment for Patients With Metastatic (Stage IV) Breast Cancer	NCT05826964	II	MBC	ER+, HER2- metastatic breast cancer	Aromatase inhibitor vs. + CDK 4/6 inhibitor versus Fulvestrant + CDK 4/6 inhibitor	United States
Fulvestrant, Ipatasertib and CDK4/6 Inhibition in Metastatic ER+/HER2- Breast Cancer Patients Without ctDNA Suppression	NCT04920708	II	MBC	ER+, HER2- metastatic breast cancer	Palbociclib and Fulvestrant and Ipatasertib vs. Palbociclib + and Fulvestrant	United Kingdom
Effect of Capivasertib on ctDNA in ER Positive Breast Cancer	NCT06613516	II	MBC	ER +/HER2- metastatic breast cancer	Cabivasertib	United Kingdom
Liquid vs. Tissue Biopsy Concordance in Samples of 1st Suspected BCa Recurrence/Metastasis and Evaluation of DefineMBC Comprehensive Cancer Profiling Liquid Biopsy LDT	NCT04962529	Observational trial	MBC	Progressive metastatic breast cancer	Tissue biopsy vs. liquid biopsy	United States
Trastuzumab Deruxtecan (T-DXd): Tailoring Treatment and Companion Diagnostics (CDx) by Liquid Biopsy	NCT05919212	Exploratory study	MBC	HER2+ metastatic breast cancer	Liquid biopsy of HER2 status	Italy
Fulvestrant and everolimus efficacy after CDK4/6 inhibitor: a prospective study with circulating tumor DNA analysis	NCT02866149	Exploratory study	MBC	ER+/HER2- metastatic breast cancer, pre-treatment with CDK 4/6 inhibitor	Fulvestrant and Everolimus	France

## Data Availability

No original data were gathered for this review.

## Abbreviations

Adjuvant therapy	AT
Allele frequency	AF
Breast imaging-reporting and data system	BIRADS
Cell-free DNA	cfDNA
Circulating tumor cells	CTCs
Circulating tumor DNA	ctDNA
Confidence interval	CI
Cyclin-dependent kinase 4/6	CDK4/6
Disease free survival	DFS
Distant disease-free survival	DDFS
Hazard ratio	HR
Minimal residual disease	MRD
Multi-cancer early detection	MCED
Neoadjuvant chemotherapy	NAT
Non-pathologic complete response	non-pCR
Overall survival	OS
Poly-ADP ribose polymerase	PARP
Polymerase chain reaction	PCR
Positive predictive value	PPV
Triple-negative breast cancer	TNBC
